# A Remarkable Coexistence of Systemic Capillary Leak Syndrome and Diabetes in an 11-Year-Old Boy: A Case Report and Review of the Literature

**DOI:** 10.1155/2020/8891902

**Published:** 2020-09-01

**Authors:** Aysun Ata, Samim Özen, Damla Gökşen, Neslihan Edeer Karaca, Güzide Aksu, Necil Kütükçüler, Hüseyin Onay, Şükran Darcan

**Affiliations:** ^1^Division of Pediatric Endocrinology, Department of Pediatrics, Ege University Faculty of Medicine, İzmir, Turkey; ^2^Division of Pediatric Immunology, Department of Pediatrics, Ege University Faculty of Medicine, İzmir, Turkey; ^3^Department of Medical Genetics, Ege University Faculty of Medicine, İzmir, Turkey

## Abstract

Systemic capillary leak syndrome (ISCLS) is a rare disease characterized by unexplained reversible capillary hyperpermeability followed by hypoperfusion, hemoconcentration, and either hypoalbuminemia or total hypoproteinemia. An 11-year-old boy was admitted with vomiting, generalized edema, and hyperglycemia, which was preceded by 5 days of coryzal symptoms, lethargy, and oral aft, without fever. On physical examination, he had tachycardia and hypotension, with severe generalized systemic nonitchy edema, and the laboratory tests supported the conclusion that he had severe hemoconcentration with hemoglobin: 184 g/L, hematocrit: 51.3 %, urea: 20 mmol/L, blood glucose: 11.1 mmol/L, and albumin: 19 gr/L, with normal urine analysis. On the fourth day, the patient was diagnosed with ISCLS, by ruling out other causes of shock and hypoalbuminemia. Intravenous immunoglobulin (IVIG) treatment regimen was administered on two consecutive days (day five and day six). His edema decreased on the fifth day, and the patient was deemed clinically well. There was no compartment syndrome, rhabdomyolysis, or pulmonary edema in the recovery period. However, respiratory virus panel PCR was positive for respiratory syncytial virus (RSV) and enterovirus, which were thought to be the triggering cause of ISCLS. For the differential diagnosis of diabetes, his fasting serum glucose was 13.4 mmol/L, simultaneous C-peptide was 0.44 nmol/L, and HbA1c was 64 mmol/mol, and urine ketone was positive. However, antiglutamic acid decarboxylase, anti-insulin antibody, and islet cell antibody were negative. At the last outpatient visit, 22 months after the diagnosis, his insulin dose was still 0.4 IU/kg/day and HbA1c was 40 mmol/mol, and without prophylaxis, there was no ISCLS attack. *Conclusion*. Early recognition of ISCLS is important for therapeutic awareness, since it is very rare in childhood and occurs usually without any prior provoking factors in healthy children. With the increase in awareness of the disease, knowledge and experiences about pediatric patients may also increase. We think that our case will contribute to the literature since there have been no pediatric diabetic patients with ISCLS reported.

## 1. Introduction

Idiopathic systemic capillary leak syndrome (ISCLS) is a rare disease characterized by unexplained reversible capillary hyperpermeability followed by hypoperfusion, hemoconcentration, and either hypoalbuminemia or total hypoproteinemia [1]. Clarkson first described a case of ISCLS in 1960 [2]. To date, about 500 cases have been reported worldwide, of which most are white adults, and it is extremely rare in children [3, 4].

Attacks of ISCLS tend to demonstrate three phases: prodromal symptoms, capillary leak, and recovery [5]. The recorded cases show that in adult patients, monoclonal gammopathy was usually accompanied, while in pediatric cases, there was no reported gammopathy [6]. For proper diagnosis of ISCLS, sepsis, anaphylaxis, and certain drug reactions need to be ruled out; there is no standard test for this diagnosis and the only way is through elimination. Here, we present a pediatric patient with the first attack of capillary leak with concordant diabetes mellitus (DM).

## 2. Case

An 11-year-old boy admitted with vomiting, generalized edema, and hyperglycemia, which was preceded by 5 days of coryzal symptoms, lethargy, and oral aft without fever. He was admitted to another hospital with hypotensive syncope and severe vomiting (20 times/day), and intravenous hydration was initiated. His blood glucose was 13.8 mmol/L and urine glucose was +++ at admission. Initially, he was diagnosed with stress hyperglycemia and acute gastroenteritis. After one day, he was referred to our hospital because of his persisting symptoms and hyperglycemia. There was no specific indicative feature in his personal or family history. His weight was 30 kg (−1.02 SDS), height was 147 cm (+0.75 SDS), heart rate was 118/minute, and blood pressure was 90/40 mm/Hg, and generalized nonitching systemic edema was present on his physical examination.

The laboratory tests showed that severe hemoconcentration was present. His biochemical findings were as follows: white blood cell count: 1.8 × 10^9^ L; hemoglobin: 184 g/L; hematocrit: 51.3%; sodium: 130 mmol/L; potassium: 4.8 mmol/L; chloride: 105 mmol/L; urea: 20 mmol/L; creatinine: 45 *µ*mol/L; and blood glucose: 11.1 mmol/L. On arterial blood gas analysis, pH was 7.33 and HCO_3_ was 13.6 mmol/L (Table 1). On sepsis work-up, CRP was 3 mg/L, and urine, stool, and blood culture were negative. Respiratory virus panel PCR was positive for respiratory syncytial virus (RSV) and enterovirus.

Aggressive fluid replacement was initiated at admission due to hypotension. Although urine output was normal during the first two days of hospitalization, his diuresis was inadequate on the third day (0.3 cc/kg/hr), and so 3000 cc/m^2^ 5% dextrose and 0.45% NaCl were administered as maintenance fluid therapy. The patient's serum albumin levels, as well as his globulin levels, were highly reduced because of capillary leak (Table 1). There was severe hypogammaglobulinemia at admission. Additional laboratory examinations were performed in order to make the differential diagnoses of generalized edema, hyperglycemia, hyponatremia, prerenal acute renal failure, hypoalbuminemia, and hemoconcentration. The urine analysis showed that the urine density was 1033, and the 24-hour urine protein test, blood lipids, and renal tubular tests were within normal limits excluding renal diseases. To eliminate congestive heart failure, chest radiography, electrocardiography, cardiac enzymes test, and echocardiography were performed and were all proven to be within normal limits. The patient's feces alpha-1 antitrypsin level was negative, and protein-losing enteropathies were excluded. Complement studies and lymphocyte subset analysis by flow cytometry were within normal ranges when compared to his age references.

**Figure 1:**
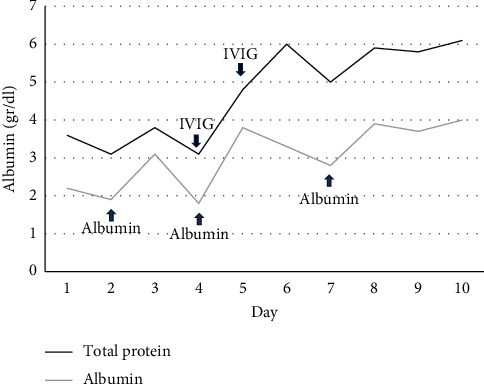
Total protein and albumin levels. 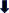: IVIG treatment 1 gr/kg. 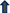: Human albumin treatment.

After initial stabilization with intravenous fluid boluses and insulin infusion the first two days, the patient's generalized edema increased, albumin values dropped to 1.9 g/dL, he had diarrhea, and he gained 3 kg of weight. Subcutaneous insulin treatment started with a dose of 0.3 IU/kg/day at the end of the second day. Intravenous human albumin treatment was given on the 2nd, 4th, and 7th day of admission. On the fourth day, the patient was given the ISCLS diagnosis by ruling out other causes. Following the diagnosis, intravenous immunoglobulin (IVIG) treatment was given on two consecutive days (Figure 1). His edema decreased on the fifth day, and he was deemed clinically well on the 7th day, weighing 29 kg. As for the systemic effects of ISCLS, there was seldom amount of free fluid in the abdomen, the diameter of the appendix was increased, and minimal pleural effusion was found bilaterally. There was no compartment syndrome, rhabdomyolysis, or pulmonary edema during the recovery period.

For the differential diagnosis of DM, his fasting serum glucose was 13.8 mmol/L, simultaneous C-peptide was 0.44 nmol/L, HbA1c was 64 mmol/mol, and urine ketone was positive. Antiglutamic acid decarboxylase, anti-insulin antibody, and islet cell antibody were negative. The patient harbors type 1 DM predisposing HLA II haplotypes as follows: DRB1∗0301, DRB1∗0302, and DRB1∗0201. Although there was no family history for DM, since his autoantibodies were negative, next-generation gene sequencing for 14 MODY genes including GCK, HNF1A, HNF4A, HNF1B, PDX1, CEL, KLF11, NEUROD1, PAX4, INS, and BLK was analyzed, and they were all negative. Whole exome sequencing of the patient for candidate genes is ongoing. At the 6^th^ and 12^th^ month marks, as well as at the last outpatient visit, his insulin dose was still 0.4 IU/kg/day and HbA1c was 40 mmol/mol.

## 3. Discussion

ISCLS is seen primarily in middle-aged adults and is rare in children, with only cases or case series being reported in children [3, 7–10]. While the general clinical features of this syndrome in children are similar to those of adults, there is no monoclonal gammopathy, and the pathogenesis and pathophysiology of ISCLS are relatively unknown due to its rarity [11]. Reports usually describe a viral prodrome and proven viral infections such as influenza virus, parainfluenza virus, enteroviruses, RSV, and rotavirus in children [7, 8, 12]. In our case, the existing prodromal symptoms and PCR results which were positive for both RSV and enterovirus suggested these viral infections had triggered the clinical findings of this case.

In its first phase, ISCLS is indistinguishable from viral infections with the symptoms of coryza, diarrhea, fatigue, and vomiting. It is this phase where patients are usually referred to a hospital, where in the second phase, the patient can be observed experiencing extravasation characterized by edema, syncope, hypotension, shock, and organ failures. This is also the critical period to diagnose and treat this disease, since if the diagnosis is given later than this stage, the mortality rate can be high as 19% due to risk of pulmonary edema, renal failure, brain edema, or shock [5].

During ISCLS flares, transient spikes in circulating angiogenic proteins, known to trigger vascular hyperpermeability (e.g., angiopoietin 2 and vascular endothelial growth factor (VEGF)), have been detected [13]. Elevated levels of cytokines and chemical mediators (granulocyte-colony stimulating factor (G-CSF), interleukin-6 (IL-6), interleukin-8 (IL-8), and monocyte chemotactic protein-1 (MCF-1)) were reported in some cases. Some authors also found elevated levels of interleukin-1*β* (IL-1*β*), C-C motif chemokine ligand-2 (CCL2), and C-X-C motif chemokine-10 (CXCL10) during the acute phase, suggesting that ISCLS may have clinically varying forms of presentation. This leads to the assumption that within the group of patients with ISCLS, different cytokines may mediate capillary leak. In an Italian girl, serum eosinophilic cationic protein was found to be elevated during acute attacks [14]. These findings demonstrate that patients with ISCLS have different cytokine profiles, which further suggests that ISCLS may consist of a heterogeneous group of disorders with the common endpoint of capillary leak [15].

The frequency and severity of episodes differ from one patient to another. Acute treatment depends on aggressive fluid replacement and crystalloid solutions [16, 17]. Corticosteroid therapy against cytokine-mediated endothelial damage along with plasmapheresis and intravenous immunoglobulin has proved to be successful in the acute phase [5, 18]. Infliximab (antitumor necrosis factor) and bevacizumab (anti-VEGF) had been used in the treatment of ISCLS attacks; however, their effects are not clear yet [19]. The usage of the IVIG treatment for two consecutive days accelerated the recovery process, parallel with what was mentioned in the literature; IVIG dramatically improves acute refractory attacks of ISCLS, but its exact mechanism remains unknown [20]. Lambert et al. reported that IVIG administration to a patient with refractory systemic capillary leak syndrome yielded dramatic improvement, and their patient was still alive 11 years after ISCLS diagnosis and receives intravenous immunoglobulins monthly [20]. Later, the authors had successfully given IVIG to two other patients during the acute phase of systemic capillary leak syndrome, and they were very well in 8 and 1.5 years of follow-up after receiving intravenous immunoglobulins at the onset of each flare.

In pediatric patients, the recurrence rate was found to be 69% [11]. Prophylactic treatment modalities are highly beneficial for recurrent attacks; the most commonly used are terbutaline, theophylline, and IVIG [21]. For prophylactic treatment of 4-year-old children, montelukast decreased attack number and severity in the follow-up [5]. In our case, we did not use prophylactic treatment, and on the 22^nd^ month of follow-up, no attacks were recorded.

To date, coexistence of DM and ISCLS is not found in the current literature. However, there are some reports claiming hyperglycemia is an independent risk factor for ISCLS in newborns [22]. Diabetic rats exhibited significant mucosal injury after 10 min of ischemia and 1 hour of reperfusion that was associated with significant capillary leak [23]. Transient hyperglycemia was reported during acute attacks of ISCLS, but the relationship between DM and ISCLS is obscure [10]. Our patient is still using 0.4 IU/kg/day insulin, on the 22^nd^ month of follow-up. Antibodies for type 1 DM were negative. The mutations for mostly known MODY genes were negative. Whether his DM made him more susceptible to ISCLS or whether the two happened to be comorbid due to an unknown etiology need to be clarified. Whole exome sequencing of patient for candidate genes is ongoing.

## 4. Conclusion

Early recognition of ISCLS is important for therapeutic awareness, since it is very rare in childhood and occurs usually without any precipitating factors in healthy children. Although there are not enough studies about acute treatment and prophylaxis, there are promising recommendations on a case basis. With the increase in awareness of the disease, knowledge and experiences about pediatric patients will also increase. We think that our case will contribute to the literature since there have been no pediatric diabetic patients with ISCLS reported.

## Figures and Tables

**Table 1:** Laboratory parameters on admission.

Test	Result	Reference values
White blood cell count (10^9^ L)	1.8 (N)	(4.5–13)
Neutrophil (10^9^ L)	1.4 (L)	(1.8–8)
Red blood cell count (10^9^ L)	6.4 (N)	(3.9–5.1)
Hemoglobin (g/L)	184 (H)	(120–150)
Hematocrit (fraction of RBC)	0.51 (H)	(0.34–0.43)
MCV (fL)	79 (N)	(76–90)
Platelet (10^9^ L)	476 (H)	(150–450)
Glucose (mmol/L)	13.6 (H)	(3.3–5.5)
Aspartate aminotransferase (*µ*kat/L)	0.35 (N)	(<0.58)
Alanine aminotransferase (*µ*kat/L)	0.15 (N)	(<0.58)
GGT (*µ*kat/L)	0.10 (N)	(<0.92)
Alkaline phosphatase (*µ*kat/L)	0.78 (L)	(2.15–6.96)
Cholesterol (mmol/L)	1.63 (N)	(<5.18)
Triglyceride (mmol/L)	2.01 (H)	(<1.69)
Sodium (mmol/L)	130.3 (L)	(136–145)
Potassium (mmol/L)	4.8 (N)	(3.5–5)
Chloride(mmol/L)	105 (N)	(96–110)
Calcium (mmol/L)	1.90 (L)	(2.15–2.50)
Phosphorus (mmol/L)	1.20 (N)	(1–1.94)
Antigliadin IgA	Negative	
Antiendomysium IgA	Negative	
Troponin T (*µ*g/L)	<13 (N)	(<14)
Creatine phosphokinase (*µ*kat/L)	0.52 (L)	(0.55–3.11)
Lactate dehydrogenase (*µ*kat/L)	2.82 (N)	(2–5)
Immunoglobulin G (g/L)	1.87 (L)	(8.22–12.8)
Immunoglobulin A (g/L)	0.43 (L)	(0.72–1.58)
Immunoglobulin M (g/L)	0.4 (L)	(0.63–1.41)
Immunoglobulin E (kU/L)	6.43 (N)	
Thyroid-stimulating hormone (mIU/L)	2.5 (N)	(0.37–4.2)
Thyroxine free (pmol/L)	19.05 (N)	(11.97–21.88)
Stool alpha-1 antitrypsin (*µ*g/g)	130 (N)	
Total protein (gr/L)	46 (L)	(64–83)
Albumin (gr/L)	22 (L)	(35–52)
Urea (mmol/L)	20 (H)	(3.57–17.85)
Creatinine (*µ*mol/L)	45.75 (N)	(22.88–76.25)
Uric acid (mmol/L)	0.35 (H)	(0.12–0.33)
C-reactive protein (mg/L)	3 (N)	(0–5)
Urine density	1033 (N)	
Urine ketone (mmol/L)	14.6 (+)	
Arterial pH	7.33 (N)	
Arterial pCO_2_ (mmHg)	25.9 (N)	
Arterial lactate (mmol/L)	1.9 (N)	
Arterial HCO_3_ (mmol/L)	13.9	
Arterial base excess (mmol/L)	−11.7	
C3 complement (g/L)	1.17 (N)	(0.9–1.8)
C4 complement (g/L)	0.15 (N)	(0.1–0.4)
Serum amyloid A (mg/L)	<3.3 (N)	
Antinuclear antibody, ANCA	Negative	
D.Coombs	+2	
C 1 esterase inhibitor (mg/L)	366 (N)	(210–390)
Antiglutamic acid decarboxylase (nmol/L)	0.01 (N)	(<0.02)
Anti-insulin antibody (nmol/L)	Negative	<0.02
Islet cell antibody	Negative	
Parvovirus B19 IgM	Negative	
Rubeola IgM	Negative	
(Varicella zoster) antibody IgM	Negative	
Antirubella IgM	Negative	
HBsAg	Negative	
EBV VCA IgM	Negative	
Anti-CMV IgM	Negative	
HSV 1 + 2 IgM	Negative	

N: normal; H: high, L:low; HbsAg: hepatitis B surface antigen; EBV VCA: Epstein–Barr virus viral capsid antigen; CMV: cytomegalovirus; HSV: herpes simplex virus; ANCA: anti-neutrophil cytoplasmic antibodies; IgM: immunoglobulin M, and IgA: immunoglobulin A.
